# Biopharmaceutical analysis — current analytical challenges, limitations, and perspectives

**DOI:** 10.1007/s00216-025-06036-2

**Published:** 2025-08-12

**Authors:** Diana R. Cunha, M. Beatriz Quinaz, Marcela A. Segundo

**Affiliations:** LAQV, REQUIMTE, Laboratory of Applied Chemistry, Department of Chemical Sciences, Faculty of Pharmacy, University of Porto, 4050-213 Porto, Portugal

**Keywords:** Biopharmaceutical analysis, Quality control, Biosimilars, Biologics, Regulatory harmonization

## Abstract

Biopharmaceutical analysis is essential for ensuring the quality, safety, and efficacy of these medicines. Nonetheless, it faces significant challenges due to the complexity of these molecules and the strict regulatory standards. This work explores the strengths, limitations, opportunities, and threats in biopharmaceutical analysis, focusing on the challenges and emerging analytical technologies and their impact on biopharmaceutical development and quality control. Key challenges identified include the complexity and heterogeneity of biopharmaceuticals leading to the requirement for a broad spectrum of analytical methods, the high cost of the required analytical instrumentation, and the need for skilled professionals to implement advanced analytical techniques. Integrating artificial intelligence-driven data analysis, automation, and multi-omics approaches can significantly improve analytical precision and efficiency. However, regulatory barriers and financial constraints remain critical obstacles to widespread adoption. This work highlights the need for industry-wide collaboration to standardize methodologies and regulations, improve data integrity, and facilitate regulatory acceptance by reviewing the last 5 years of biopharmaceutical-related works. Ultimately, the continued development of biopharmaceutical analysis, driven by technological advancements and strategic investments, is crucial for biopharmaceutical development, enhancing manufacturing efficiency, and ensuring broader access to high-quality biopharmaceuticals.

**Graphical Abstract:**

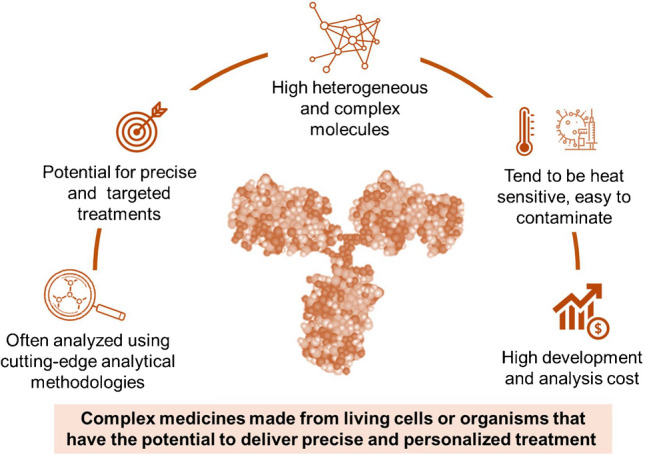

## Introduction

**Fig. 1:**
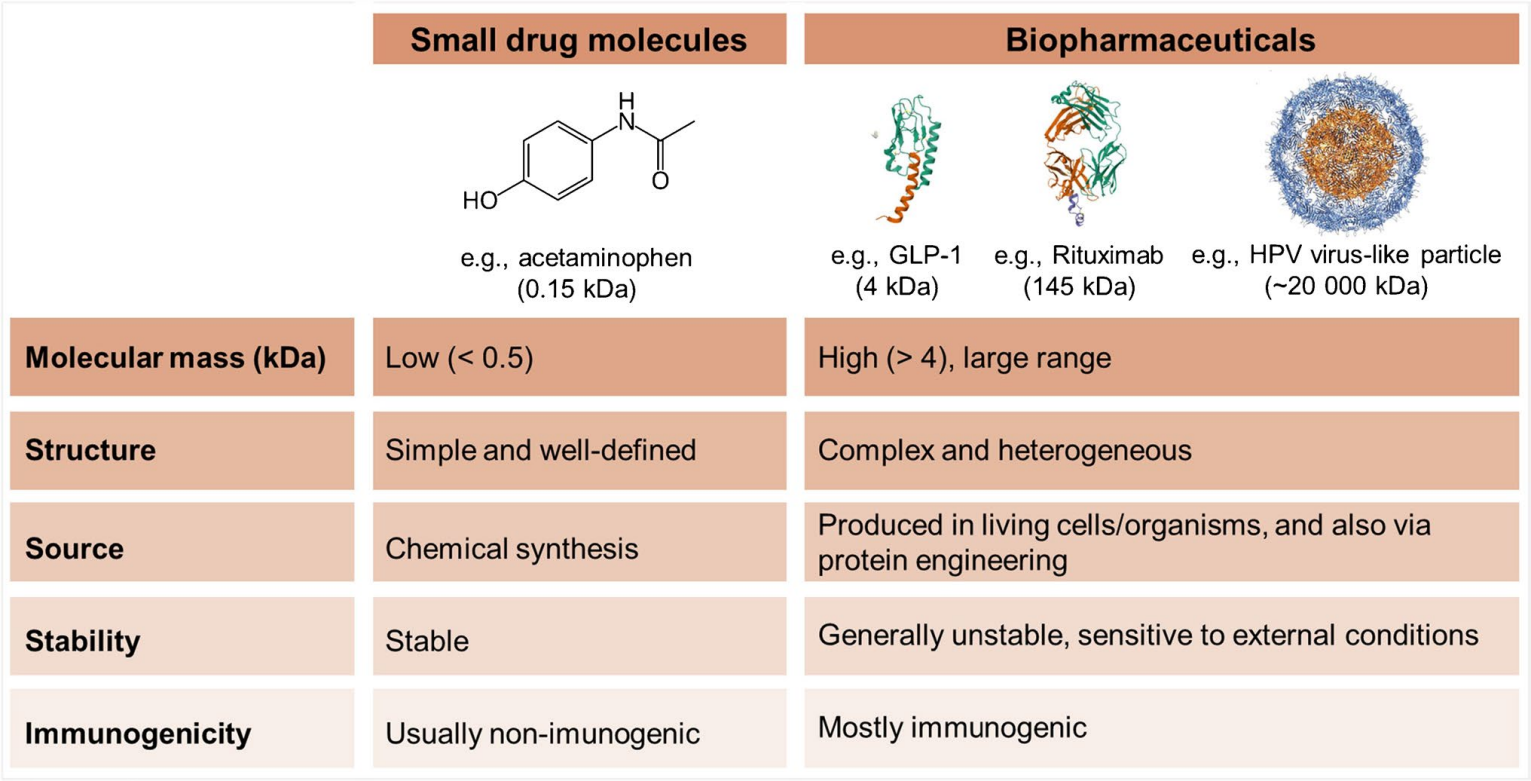
Key structural and functional differences between small traditional drug molecules and biopharmaceuticals (GLP-1, glucagon-like peptide-1)

Biopharmaceuticals, also referred to as biologics, represent a class of therapeutic agents derived from biological systems or developed through advanced protein engineering. This family of therapeutic agents comprises a diverse range of molecules, including recombinant proteins, peptides, nucleic acids, and cell-based therapies, which have significantly transformed modern medicine by enabling precise and targeted treatment strategies for a wide range of diseases, including autoimmune disorders, cancers, infectious diseases, and genetic conditions [1, 2]. Monoclonal antibodies (mAbs), for example, have revolutionized oncology by facilitating precision medicine approaches, while recombinant proteins and gene therapies have introduced novel treatment options for rare and previously untreatable genetic disorders [3–6]. Due to their structural complexity and sensitivity to environmental factors, biopharmaceuticals require specialized care during development, production, storage, and transportation, posing considerable challenges in their manufacturing and distribution. Beyond their clinical impact, biopharmaceuticals contribute to healthcare sustainability by reducing healthcare costs associated with long-term disease management and hospitalization [2, 7, 8]. In contrast to conventional small-molecule drugs, biopharmaceuticals are characterized by high molecular weight, complex and heterogeneous structures, and advanced manufacturing processes. These attributes contribute to their enhanced target specificity, improved clinical efficacy, and safety profiles. However, biologics are inherently more susceptible to degradation, immunogenic responses, and stability concerns compared to small drug molecules (Fig. 1) [8].

The global biopharmaceutical market has experienced rapid growth over the past decade, driven by advancements in biotechnology, increased investments in research and development (R&D), linked to an aging population, and the growing demand for targeted and innovative therapies. Market analysis estimates that the global biopharmaceutical sector was valued at approximately USD 452 billion in 2024, with projections suggesting it will reach USD 484 billion by 2025. Among biologic therapies, mAbs dominate the market, accounting for 61% of total revenue in 2024, largely due to their broad therapeutic applications. The biopharmaceutical industry is expected to sustain its upward trajectory, with a projected compound annual growth rate (CAGR) of 8.87% from 2025 to 2030, reaching an estimated market size of USD 740 billion by 2030 [9]. These growth projections highlight the sector’s robust expansion potential, driven by continuous innovation, strategic investments, and an expanding pipeline of biologic therapeutics. However, challenges such as regulatory uncertainties, geopolitical influences, and societal shifts may impact market dynamics and growth trajectories.

**Fig. 2:**
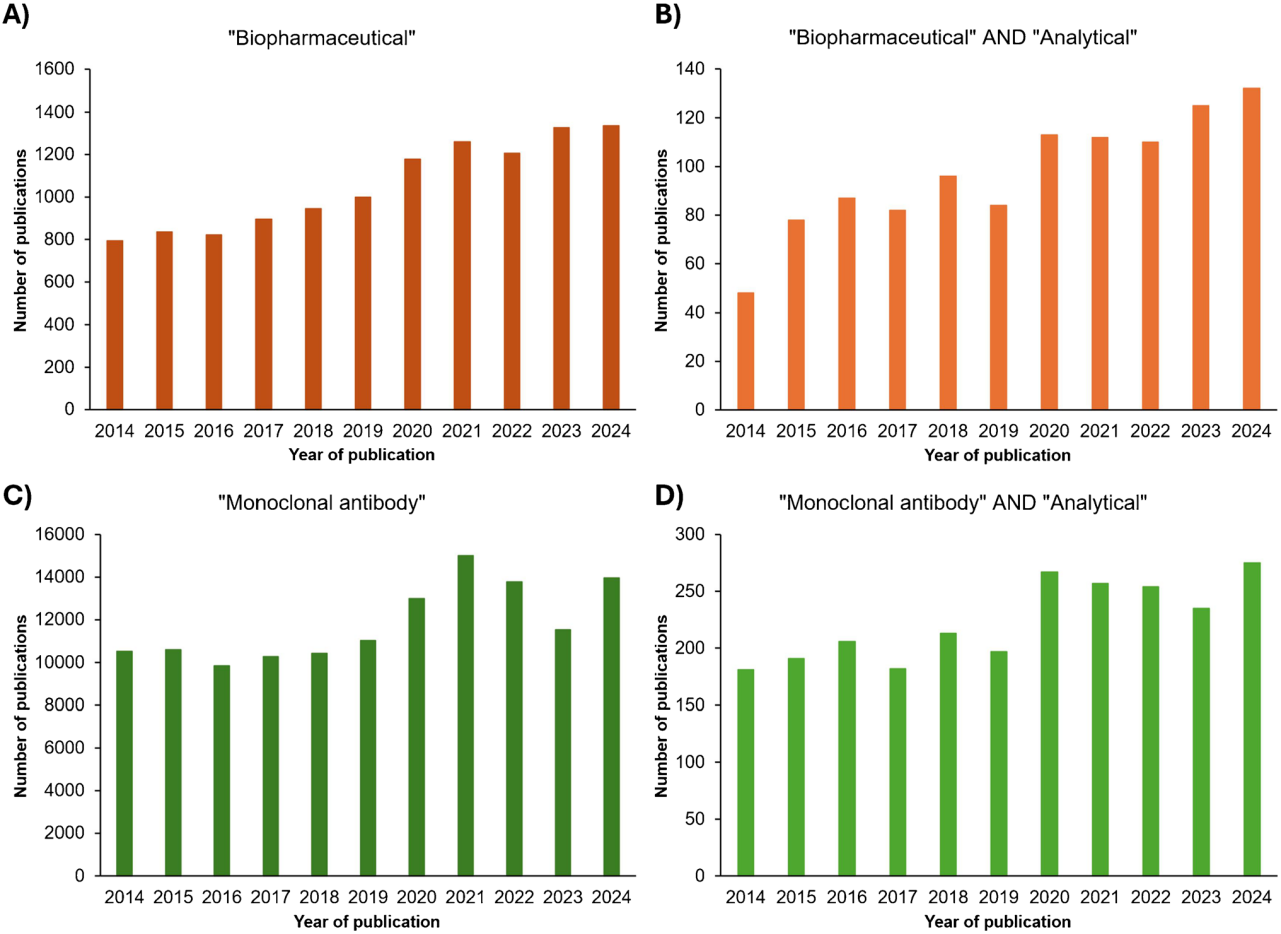
Publication trends related to biopharmaceuticals and monoclonal antibodies from 2014 to 2024, based on Scopus data. The graph presents **A** total publications containing the keyword “biopharmaceuticals”; **B** publications containing both “biopharmaceuticals” and “analytical”; **C** total publications containing “monoclonal antibody”; and **D** publications containing both “monoclonal antibody” and “analytical”

In parallel with market expansion, the field of biopharmaceutical analysis has demonstrated substantial growth, as evidenced by the increasing number of related scientific publications (Fig. 2). According to data retrieved from Scopus, publications containing the keyword “biopharmaceuticals” increased by approximately 64%, rising from 794 in 2014 to over 1,300 by 2024. More specifically, publications that include both “biopharmaceutical” and “analytical” terms increased about 171%, from 48 to over 130 in the same period. A similar upward trend is observed for “monoclonal antibody,” which rose from approximately 10,500 publications in 2014 to over 13,900 in 2024, representing a 32% increase. Publications that combine “monoclonal antibody” with “analytical” grew from 181 to 275, marking a 52% increase. These metrics clearly illustrate the expanding interest and ongoing innovation in analytical strategies for biopharmaceuticals, underscoring the relevance of a critical review focused on current challenges, limitations, and emerging perspectives in this rapidly advancing field.

Despite the remarkable advancements in biopharmaceutical development, their structural complexity and physicochemical heterogeneity present substantial analytical challenges that require sophisticated characterization and quality control (QC) methodologies. Ensuring the consistency, efficacy, and safety of biologics requires highly sensitive and robust analytical techniques. Unlike small drug molecules, which can be characterized using well-established chromatographic and spectrometric techniques, biologics require an integrated approach combining multiple orthogonal analytical methodologies [10, 11].

The rigorous characterization is particularly critical for both originator biologics (OB) and biosimilars. Biosimilars are biologic products designed to be highly similar to an approved reference biologic, with no clinically meaningful differences in safety, purity, or potency. As patents for OBs expire, biosimilars present an opportunity to expand patient access to biologic therapies while reducing healthcare costs. Regulatory agencies, such as the U.S. Food and Drug Administration (FDA) and the European Medicines Agency (EMA), have established stringent guidelines for biosimilar approval, emphasizing the need for robust analytical frameworks to detect potential variations that could impact therapeutic performance. The biosimilars market has experienced significant growth in recent years, driven by the increasing prevalence of chronic diseases, the demand for cost-effective therapies, and the expiration of patents for major OB drugs [12, 13]. In 2022, the global biosimilars market was valued at approximately USD 21.8 billion and is projected to reach USD 76.2 billion by 2030, reflecting a CAGR of 15.9% during the forecast period [14].

While biopharmaceuticals have transformed modern therapies, their analytical complexity requires the development of advanced, multidimensional characterization methodologies. The growing prominence of biosimilars further highlights the need for rigorous analytical frameworks. Several comprehensive works have examined key aspects of biopharmaceutical analysis. Some have provided valuable overviews of emerging analytical tools, particularly advanced chromatographic techniques [15], electrophoresis by imaged capillary isoelectric focusing [16], and other techniques in the context of process analytical technology [17], with applications in quality control and process monitoring. Others have focused on regulatory frameworks and quality standards [18], as well as the growing impact of machine learning in enhancing the efficiency of biopharmaceutical development and production [19]. While these works have significantly contributed to the field by addressing specific analytical techniques or isolated aspects of method development, the present review offers a broader and more integrated perspective. It synthesizes current analytical challenges, limitations, and innovations across the biopharmaceutical lifecycle, offering a comprehensive and holistic understanding of the field. It explores key methodologies employed for structural and functional characterization, the regulatory landscape for biosimilars, and potential innovations to address existing analytical gaps in biopharmaceutical analysis.

## Current challenges in biopharmaceutical analysis

### Diversity of molecular structures

Biopharmaceuticals hold a broad range of molecules including, recombinant proteins, mAbs, gene and cell-based therapeutics, vaccines, and tissue-engineered therapeutics (Table 1). Their structural complexity and inherent heterogeneity present significant analytical challenges, impacting both characterization and regulatory compliance. This heterogeneity arises from multiple factors, including variations in molecular size, ranging from approximately 4000 nucleotides (e.g., gene therapies), and 150 kDa (e.g., recombinant proteins and mAbs) to as large as 20,000 kDa (e.g., virus-like particles—a self-assembling nanoparticle, derived from viral structural proteins, that precisely mimics the outer architecture of a native virus but entirely lacks the infectious genetic material, making it a safe and highly immunogenic platform for vaccines, drug delivery, and gene therapy). Moreover, protein-based biopharmaceuticals exhibit intricate folding patterns and HOS due to their secondary, tertiary, and quaternary conformations, further introducing complexity to their analysis. This complexity stems from batch-to-batch variability inherent to their production in specific and diverse expression systems (e.g., *Escherichia coli*, *Saccharomyces cerevisiae*, and Chinese hamster ovary (CHO) cells), as well as various post-translational modifications (PTM), such as glycosylation (e.g., in monoclonal antibodies) and disulfide bond formation (e.g., in insulin), introducing additional structural variations.

Biopharmaceuticals are also susceptible to microheterogeneities, which may arise intrinsically or due to chemical and enzymatic modifications during manufacturing, storage, or interactions with host cell proteins [20–22]. Comprehensive analysis of biopharmaceuticals often requires the integration of orthogonal techniques to achieve accurate and detailed structural elucidation. Unlike small molecules, which benefit from well-established standardized analytical protocols, biopharmaceuticals require tailored approaches due to their inherent structural diversity. The absence of standardized analytical protocols across different laboratories and industries hampers consistency and reproducibility. This lack of harmonization, combined with the need for extensive method validation, ultimately impacts the speed-to-market of biopharmaceutical products, delaying their clinical availability [11, 23]. Furthermore, the continuous emergence of novel biopharmaceuticals requires ongoing innovation in analytical techniques, as traditional methods often fall short of providing suitable comprehensive characterization.

**Table 1:** Types of biopharmaceuticals and main features

Biopharmaceutical type	Examples	Clinical indication	Production	Size
Recombinant proteins	Insulin glargine	Diabetes	Recombinant DNA technology in *Escherichia coli*	5.7 kDa
	Etanercept	Autoimmune conditions such as rheumatoid arthritis	Recombinant DNA technology in CHO mammalian cells	150 kDa
	Glucagon-like peptide-1 (GLP-1)	Type 2 diabetes mellitus	Recombinant DNA technology in *Saccharomyces cerevisiae*	4 kDa
	Epoetin alfa	Symptomatic anemia	Recombinant DNA technology in CHO mammalian cells	30 kDa
Monoclonal antibodies (mAbs)	Rituximab	Lymphoma non-Hodgkin;	Recombinant DNA technology in CHO mammalian cells	145 kDa
	Fremenazumab	Migraine	Recombinant DNA technology in CHO mammalian cells	145 kDa
Vaccines	Tozinameran (mRNA)	SARS-CoV-2	Cell-free in vitro transcription of nucleotide sequence encoding the viral spike (S) protein of SARS-CoV-2 from the corresponding DNA templates	141 kDa
Gene Therapy Products	Voretigene neparvovec	Retinal dystrophy	Recombinant DNA technology in an adeno-associated viral vector serotype 2 (AAV2)	4700 base pairs
	Onasemnogene abeparvovec	Spinal muscular atrophy	Recombinant DNA technology in human embryonic kidney cells	4600 base pairs
Cell Therapy Products	CAR-T cell therapy	Blood cancer	Genetically engineering a patient’s own T cells	-
Tissue-engineered Products	Holoclar	Limbal stem cell deficiency	Ex vivo expanded autologous human corneal epithelial cells containing stem cells	-

*CHO*, Chinese hamster ovary

### Quality control

Developing robust, reproducible, environmentally sustainable, and high-throughput analytical methods for biopharmaceutical QC remains a significant challenge. The selection of appropriate QC methods requires careful evaluation of sensitivity, specificity, regulatory compliance, and practical feasibility [20, 24]. QC approaches vary considerably between hospital pharmacy settings (point-of-care) and industrial manufacturing. While hospital QC is less exhaustive than industrial manufacturing, both play a critical role in preserving the integrity of biopharmaceuticals from production to patient administration.

In hospital pharmacies, QC is needed during biopharmaceutical preparation, storage, and administration to ensure patient safety. Key analytical aspects include verifying reconstitution and dilution accuracy, to confirm the correct preparation of biopharmaceuticals (e.g., monoclonal antibodies or personalized cell therapies) in accordance with manufacturer guidelines [22, 25, 26].

Most analytical methods commonly used for biopharmaceutical quantification and identification, including capillary electrophoresis (CE), enzyme-linked immunosorbent assays (ELISA), and liquid chromatography-mass spectrometry (LC–MS), are not well-suited for routine hospital QC [22]. These methods often require complex sample preparation, are time-consuming, and involve high operational costs, making them impractical for rapid, point-of-care analysis. Spectroscopy-based methods have emerged as more viable methodologies for hospital QC due to their rapid analysis, simplicity, and minimal sample preparation requirements. Instruments incorporating Ultraviolet/Infrared (UV/IR) and UV/Raman spectroscopy, such as Multispec® and QC Prep + ®, have been designed for drug QC. Additionally, flow injection analysis (FIA) combined with UV spectroscopy has been proposed as a rapid and efficient approach for the identification and quantification of mAbs, offering a practical solution for point-of-care QC in hospital pharmacy [25, 27–31].

In contrast, industrial biopharmaceutical QC is highly structured and strictly regulated by agencies such as the FDA, the EMA, and the International Council for Harmonisation (ICH). This process involves comprehensive analytical testing, requiring specialized facilities, automated systems, and rigorous validation protocols to ensure batch-to-batch consistency before commercial distribution. Several analytical methods have been employed to assess biopharmaceuticals’ key attributes (Table 2) including identity, purity, potency, structural integrity, aggregation, stability, glycosylation, and PTMs. Each method offers distinct advantages and limitations, making some of them more suitable than others for specific applications. No single method is sufficient on its own, requiring a multi-technique approach for comprehensive characterization.

Advanced analytical methods, including native mass spectrometry (HRMS), nuclear magnetic resonance (NMR) spectroscopy, and peptide mapping (typically performed by LC–MS/MS), are widely employed for identity confirmation and structural characterization of biopharmaceuticals. Among these, MS, particularly LC–MS, is considered a gold standard in biopharmaceutical analysis due to its precision in determining molecular weight and sequence through the analysis of digested peptides. Peptide mapping, often combined with chromatographic separation, provides detailed insights into the primary structure and PTMs. However, both MS and peptide mapping require specialized instrumentation and expertise, with complex data interpretation. While NMR spectroscopy offers atomic-level resolution, its application in biopharmaceutical analysis is limited by high costs and lower sensitivity compared to MS, significant spectral overlap and complexity inherent to large molecular, and the frequent requirement for isotopic labeling [11, 32–36].

Ensuring biopharmaceuticals purity is essential for minimizing immunogenic responses. These therapeutics exhibit a complex impurity profile, including residual protein A, host cell proteins (HCP), host cell DNA, and other contaminants from cell culture or purification processes. A comprehensive impurity assessment requires a combination of orthogonal analytical methods [22, 37]. Chromatographic and electrophoretic techniques, such as high-performance liquid chromatography (HPLC) and CE, coupled with suitable detectors, are widely recommended for impurity analysis. LC, using size-exclusion (SEC) [38, 39], ion-exchange (IEX) [40], and reversed-phase (RP) [41, 42] approaches, offers high specificity and efficiency but requires extensive method development [43]. CE provides high-resolution separation of charge variants and glycoforms [44–47]. Additional methods, such as sodium dodecyl sulfate–polyacrylamide gel electrophoresis (SDS-PAGE) and biosensors, support routine impurity detection. While SDS-PAGE is simple and cost-effective, its limited quantification capability and resolution restrict its utility. Integrating SDS with CE has been shown to enhance sensitivity, precision, and speed for separating biomolecules of variable size [48]. Biosensors enable real-time impurity detection but may lack the robustness and specificity of chromatographic techniques [22, 49]. In impurity analysis, differentiating subtle variations from the primary biologic critically relies on separative methods (e.g., HPLC). These techniques are essential because many impurities, including product-related variants and degradation products, often share physicochemical properties or reactivity profiles highly similar to the target biologic. This inherent similarity limits the reliable application of non-separative quantification methods, as they cannot effectively distinguish the biopharmaceutical from protein-based impurities.

Assessing the potency and biological activity of biopharmaceuticals is essential for ensuring therapeutic efficacy reflecting the drug biological activity in clinical situations [22]. ELISA, cell-based assays, surface plasmon resonance (SPR), and biosensors are widely employed for this purpose. ELISA offers high specificity and sensitivity but is susceptible to cross-reactivity and limited to known antigens and antibodies [50–52]. Cell-based assays evaluate functional responses such as receptor binding and signal transduction, providing a direct measure of bioactivity, though they are time-consuming and prone to variability [53]. SPR and biosensors enable real-time biomolecular interaction analysis, yielding valuable insights into binding affinities [54–58].

Accurate quantification of biopharmaceutical concentration is also essential for potency assessment and formulation development. UV–Vis spectroscopy (A280, A260/A280) is a widely used, rapid, and non-destructive technique for biopharmaceutical quantification. However, its accuracy can be compromised by the presence of contaminants that interfere with absorbance measurements, leading to errors in protein concentration. Therefore, due to its non-specificity and susceptibility to interference from impurities like nucleic acids and host cell components, UV–Vis spectroscopy is typically recommended for use only in the final steps of quality control, when samples are highly purified and interference is minimized [59, 60]. The assessment of a biopharmaceutical’s efficacy critically relies on methods employing molecular recognition (e.g., ELISA and SPR). These assays, which rely on specific binding interactions, are essential for evaluating not just the quantity, but the bioactivity and functional integrity of the therapeutic molecule by mimicking its interaction with biological targets. Characterizing the three-dimensional structure of biopharmaceuticals is fundamental for assessing stability and functionality. X-ray crystallography, circular dichroism (CD), and Fourier transform infrared (FTIR) spectroscopy are commonly used for structural characterization. X-ray crystallography provides atomic-resolution structures, but not all biopharmaceuticals crystallize, limiting its applicability. CD and FTIR offer rapid and non-destructive secondary structure analysis; still, they provide less detailed structural information compared to crystallography [61–66]. Dynamic light scattering (DLS), size-exclusion chromatography coupled with multi-angle light scattering (SEC-MALS), and analytical ultracentrifugation (AUC) are key techniques for biopharmaceutical aggregation analysis. DLS enables rapid, non-destructive detection of particle size distribution but has limited sensitivity for small aggregates [67]. In contrast, SEC-MALS offers precise molecular weight determination and quantification of aggregates, making it a widely accepted method despite potential artifact formation [38, 68]. Moreover, AUC, as a quantitative technique, provides high-resolution analysis of molecular weight, size distribution, and interactions in native solution conditions without the need for sample treatment. While it offers advantages such as eliminating the need for reference standards and accommodating a wide range of particle sizes, it requires specialized expertise and extensive data analysis [69, 70]. For stability and degradation studies, differential scanning calorimetry (DSC) and LC–MS are widely used, playing a crucial role in assessing shelf-life and formulation robustness. DSC measures thermal stability by detecting unfolding protein events, offering valuable insights into conformational changes. However, it requires large amounts of sample (hundreds of micrograms to milligrams) and is not well-suited for high-throughput screening [71]. LC–MS enables precise identification of degradation products, such as oxidation and deamidation, but is associated with high costs and extensive method optimization challenges, complex data interpretation for intact or digested molecules, strict sample preparation requirements, and potential limitations in detecting very low-abundance species [32, 33, 35, 64].

LC–MS and hydrophilic interaction liquid chromatography-mass spectrometry (HILIC-MS) are extensively employed for glycosylation and PTM analysis, both of which significantly impact biopharmaceutical function, stability, and safety. These methods enable high-resolution glycan profiling and the simultaneous detection of multiple PTMs, making them indispensable for regulatory compliance. However, their application requires advanced analytical expertise, sophisticated instrumentation, and extensive data processing capabilities. Additionally, derivatization reactions are often necessary to enhance ionization, improve chromatographic separation, and stabilize glycan structures, further adding to the complexity and environmental impact [33, 35, 72, 73].

Next-generation sequencing (NGS) has recently emerged as an indispensable tool in the QC of biopharmaceuticals, offering extensive information in genetic characterization and biosafety assessment. Unlike traditional methods that provide limited sequence information, NGS enables comprehensive analysis of the entire production system, from the host cell line to the final product. Its primary application in QC lies in verifying the genetic stability and identity of the cell line, performing highly sensitive and unbiased screening for residual host cell DNA and adventitious agents (e.g., viruses, bacteria, and fungus). This capability is important for confirming product safety, particularly as regulatory expectations evolve towards more exhaustive contaminant detection. However, the integration of NGS into routine QC workflows presents challenges, including the need for robust bioinformatics expertise to manage and interpret huge datasets, and the high initial investment in instrumentation. Despite these complexities, the incomparable specificity and sensitivity of NGS addresses critical analytical limitations and offers a forward-looking perspective on ensuring the quality, safety, and consistency of biopharmaceutical products [74, 75].

**Table 2:** Analytical methods applied to characterization of biopharmaceuticals

Analytical purpose	Method	Key features	Quantitative/qualitative	Pros	Cons	Applicable biopharmaceuticals	Ref
Identity testing	MS	Determines molecular weight and sequence with high precision	Qualitative and quantitative	- High specificity and sensitivity - Suitable for complex mixtures	- Requires specialized equipment and expertise - Data interpretation and sample preparation can be complex	Recombinant proteins; Peptides; mAbs; Fusion proteins; Antibody–drug conjugates	[11, 32, 33]
	Peptide mapping	Enzymatic digestion followed by chromatographic separation to assign the primary structure	Qualitative	- Detailed structural information - Effective for detecting sequence variants	- Time-consuming sample preparation - Dependent on enzyme specificity - Interpretation can be challenging	Recombinant proteins; mAbs; Fusion proteins; Antibody–drug conjugates;	[34, 35]
	NMR	Provides information on molecular structure and dynamics	Qualitative	- Non-destructive - Offers detailed structural insights	- Low sensitivity - Requires large sample quantities (mg) - Expensive instrumentation	Smaller recombinant proteins; Peptides; Nucleic acids	[36]
Purity, quantity, and impurity analysis	HPLC	Separates components based on interactions with the stationary/mobile phase	Quantitative	- High resolution - Easily adaptable to several detectors	- Method development can be time-consuming - Requires pure standards for calibration - Potential for matrix effects	Recombinant proteins; mAbs; Peptides; Nucleic acids	[22, 43]
	CE	Separates molecules based on their size-to-charge ratio in an electric field	Quantitative	- High efficiency - Requires small sample volumes - Suitable for charged species	- Sensitive to sample matrix - Require complex method development - Often require specialized capillaries or additives	Smaller recombinant proteins; Peptides; Nucleic acids	[44–47]
	SDS-PAGE	Separates proteins based on molecular weight under denaturing conditions	Qualitative and semi-quantitative	- Simple and cost-effective - Enables protein purity and molecular weight analysis	- Limited quantification - Lower resolution compared to chromatographic methods - Manual steps can impact reproducibility	Recombinant proteins; mAbs; Peptides	[22]
	Biosensors (e.g., Immunosensors)	Detect specific interactions between biomolecules and monitor real-time binding events	Quantitative	- High sensitivity - Real-time and rapid analysis - Minimal sample preparation	- Potential for non-specific binding - Limited multiplexing capabilities - Sensor surface fouling	Recombinant proteins; mAbs; peptides; nucleic acids; cells; virus-like particles	[22, 54, 57, 58]
	UV–Vis spectroscopy (A260, A280)	Measures protein concentration based on intrinsic light absorption	Quantitative	- Rapid - Non-destructive - Requires minimal sample preparation	- Interference from UV-absorbing compounds - Non-specific - Not suitable for impurity analysis	Recombinant proteins; mAbs; Nucleic acids	[22, 59, 60]
Potency, quantity, and bioactivity	ELISA	Detects and quantifies specific proteins or antibodies using antigen–antibody interactions	Quantitative	- High sensitivity and specificity - Suitable for various analytes	- Potential for cross-reactivity - Limited to known antigens or antibodies - Requires high-quality antibodies - Often labor-intensive for manual formats	Recombinant proteins; mAbs; Vaccines; Peptides	[22, 50, 51]
	Electrochemical and optical biosensors	Measure biological interactions through changes in electrical or optical signals	Quantitative	- Rapid - Real-time analysis - Miniaturization is possible	- May require complex fabrication - Potential interference from sample matrix	Recombinant proteins; mAbs; peptides; nucleic acids	[22, 54, 57, 58]
	Cell-based assays	Evaluate biological response of living cells to a biopharmaceutical	Quantitative	- It can assess multiple pathways - Reflects the drug’s mechanism of action	- High variability - Complex to standardize - Time-consuming and resource-intensive	Recombinant growth factors; mAbs; Recombinant cytokines; Vaccines	[22, 51]
	SPR	Monitors real-time binding interactions	Quantitative	- Label-free detection - Provides kinetic data - Minimal sample preparation - Real-time monitoring	- Requires dedicated and expensive instrumentation - Sensitive to temperature and refractive index changes - Requires purified samples	Recombinant proteins; mAbs; peptides; nucleic acids; cells; virus-like particles	[22, 55]
Structural Characterization	X-ray Crystallography	Determines atomic-resolution 3D detailed structural information	Qualitative	- High-resolution structural data	- Requires successful crystallization - Time-consuming - Provides only a static snapshot of the structure	Recombinant proteins; Vaccines; Nucleic acids	[76, 77]
	CD	Assesses protein secondary structure	Qualitative	- Rapid analysis - Requires small sample amount - Non-destructive - Minimal sample preparation	- Limited structural information - Low resolution - Overlapping absorption bands can complicate analysis	Recombinant proteins; mAbs; Peptides; Nucleic acids	[22, 63, 64]
	FTIR	Analyzes secondary structure by infrared absorption	Qualitative	- Non-destructive - Rapid analysis	- Overlapping absorption bands can complicate the analysis - Lower sensitive	Recombinant proteins; mAbs; Peptides; Nucleic acids	[64–66]
Aggregation, stability and degradation studies	DLS	Measures particle size distribution in solution by analyzing light scattering patterns	Quantitative	- Rapid analysis - Non-destructive - Requires minimal sample preparation	- Limited sensitivity for small aggregates - Less effective for low-concentration samples	Recombinant proteins; mAbs; Nucleic acids	[22, 67]
	SEC-MALS	Separates molecules based on size; MALS provides absolute molecular weight determination	Quantitative	- High-resolution separation - Accurate molecular weight determination - Effective for detecting aggregates	- Requires careful column calibration and robust method development - Potential for artifacts	Recombinant proteins; mAbs	[22, 38]
	AUC	Measures sedimentation behavior of macromolecules under high centrifugal forces	Quantitative	- High-resolution analysis of aggregates - Suitable for a wide range of particle sizes - Does not require reference standards	- Requires specialized equipment and expertise - Time-consuming data analysis - Low throughput	Recombinant proteins; mAbs; nucleic acids; Virus-like particles	[69, 70]
	DSC	Measures heat changes associated with protein unfolding to assess thermal stability	Quantitative	- Provides precise melting temperature (Tm) - Non-destructive - Requires no labeling	- Requires relatively large sample quantities (mg) - Not suitable for high-throughput screening	Recombinant proteins; mAbs; Peptides; Nucleic acids	[64, 71]
	LC-HRMS	Combines separation capabilities of liquid chromatography with mass analysis for detailed characterization	Quantitative	- High sensitivity and specificity - Capable of identifying and quantifying degradation products	- Requires specialized equipment and expertise - Potential ionization suppression effects - Extensive and complex sample preparation	Recombinant proteins; mAbs; Fusion proteins; Peptides	[32, 33, 35]
Glycosylation and PTMs	LC–MS	Detailed analysis of glycan structures and other PTMs; capable of site-specific characterization	Quantitative	- High resolution and sensitivity - Allows for comprehensive profiling of glycosylation patterns - Detects multiple PTMs simultaneously	- Complex data interpretation - Requires specialized equipment - Sample preparation complexity -Need for highly skilled personnel	Glycoproteins, monoclonal antibodies	[11, 32, 33, 35]
	HILIC-MS	Separate glycans based on hydrophilicity; coupled with MS for detailed structural information	Quantitative	- Effective for separating isomeric glycans	- Requires derivatization of glycans - Method development can be complex	Glycoproteins, monoclonal antibodies	[22, 33, 35, 73]
Genetic characterization and biosafety	NGS	Sequencing of DNA or RNA fragments	Qualitative and quantitative	-Comprehensive impurity profiling - Detection of subtle genetic variations - Cell line characterization - High throughput	- Requires bioinformatics expertise - Complex sample preparation - Does not directly assess protein structure/function	Applicable to all biologics	[74, 75]

*AUC*, analytical ultracentrifugation; *CD*, circular dichroism; *CE*, capillary electrophoresis; *DSC*, differential scanning calorimetry; *DLS*, dynamic light scattering; *ELISA*, enzyme-linked immunosorbent assay; *FTIR*, Fourier transform infrared; *HILIC-MS*, hydrophilic interaction liquid chromatography-mass spectrometry; *HPLC*, high-performance liquid chromatography; *LC–MS*, liquid chromatography-mass spectrometry; *mAbs*, monoclonal antibodies; *MS*, mass spectrometry; *NGS*, next-generation sequencing; *NMR*, nuclear magnetic resonance; *PTMs*, post-translational modifications; *SDS-PAGE*, sodium dodecyl sulfate–polyacrylamide gel electrophoresis; *SEC-MALS*, size-exclusion chromatography with multi-angle light scattering; *SPR*, surface plasmon resonance; *UV–Vis*, ultraviolet–visible

### Biosimilarity assessment

**Fig. 3:**
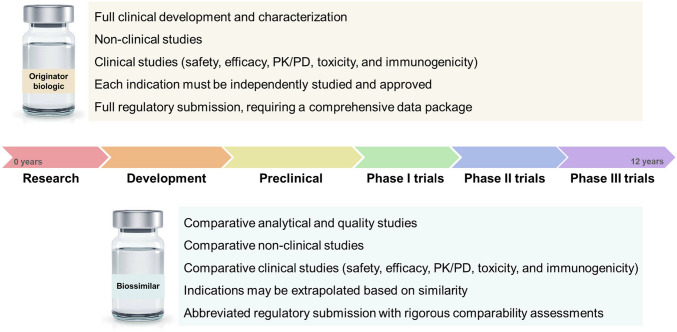
Comparative overview of biosimilarity assessment process

With the increasing development of biosimilars, assessing their biosimilarity to the OB presents a significant challenge due to the inherent complexity and heterogeneity of these molecules (Fig. 3) Establishing clear biosimilarity criteria is difficult, thereby increasing the relevance of regulatory and analytical aspects [78]. Demonstrating biosimilarity requires a comprehensive analytical characterization of physicochemical and structural attributes, including amino acid sequences, PTMs, and HOS. Biopharmaceuticals, such as mAbs and fusion proteins, exist as mixtures of isoforms arising from PTMs, but current analytical methodologies often struggle to fully characterize these molecular variants. For example, distinguishing isomeric amino acids, such as leucine and isoleucine, remains a persistent analytical challenge [78–81]. A critical obstacle in biosimilarity assessment is the characterization of HOS and protein aggregation. While techniques such as X-ray crystallography and NMR spectroscopy provide structural insights, their routine application is often impractical due to time constraints and sensitivity limitations. Furthermore, in the case of protein aggregation monitoring, widely used analytical techniques, such as SEC and AUC, exhibit limitations in detecting certain aggregate sizes (e.g., low-order oligomers and very large insoluble aggregates) [80, 81].

Another major challenge lies in the selection and sourcing of reference materials. Analytical methodologies must be sufficiently sensitive to detect minor differences between the biosimilar and the OB. However, the isolation of active pharmaceutical ingredients from OBs, which are typically available only as formulated drug products, involves complex extraction processes that may change their properties, complicating direct comparability assessments [81, 82]. Since biosimilar manufacturers do not have access to proprietary manufacturing details of the OB (e.g., genetically engineered host cell line characteristics, specific upstream cell culture conditions, or downstream purification protocols), they must employ reverse engineering to develop a highly similar product. Nevertheless, even minor differences in manufacturing conditions can result in variations in glycosylation patterns and other critical quality attributes, making the assessment of biosimilarity difficult [12]. Regulatory considerations further increase the complexity of biosimilarity evaluations. Different regulatory agencies, such as the FDA and the EMA, employ distinct terminologies and criteria for biosimilarity assessment, such as “similar” vs. “highly similar.” Establishing acceptable levels of comparability is particularly challenging when evaluating immunogenicity, where assay sensitivity and drug tolerance are critical determinants [12, 79]. Despite advancements in analytical instrumentation, challenges persist in establishing standardized methods that ensure accurate, reproducible, and regulatory-compliant data. Addressing these obstacles requires a multifaceted approach that integrates state-of-the-art analytical techniques with robust statistical evaluations to demonstrate biosimilarity with high confidence [12, 78, 79, 82].

Currently, a wide range of advanced analytical techniques are employed for the evaluation of key physicochemical properties of the biosimilar (e.g., primary structure, HOS, PTMs, charge heterogeneity, and aggregation) and biological activity (e.g., biological response, receptor binding, cell proliferation, cytotoxicity, and immunogenicity). Impurity profiling (process-related impurities, degradation products, contaminants) and stability studies under various storage conditions are also critical to ensure long-term product quality, safety, and efficacy. Mass spectrometry (e.g., intact mass analysis, peptide mapping) is crucial for detailed structural elucidation and for identifying modifications [83–85]. Chromatographic methods such as SEC, IEX, and RPLC are used to assess molecular size, charge variants, aggregation, and purity [86–89]. Spectroscopic techniques like CD and FTIR spectroscopy provide insights into HOS and conformational integrity [90, 91]. CE techniques, including capillary isoelectric focusing (cIEF) and capillary gel electrophoresis (CGE), are also vital for assessing charge heterogeneity and molecular size [92, 93]. Additionally, the in vitro bioassays are designed to confirm that the biosimilar elicits comparable biological responses to the OB [94]. Collectively, these comprehensive analytical and functional data establish the base for demonstrating a high degree of similarity.

### Physiological barriers and drug delivery challenges

While most biopharmaceuticals have traditionally been administered via parenteral routes (e.g., intravenous and subcutaneous) due to their susceptibility to enzymatic degradation in the gastrointestinal tract and limited membrane permeability, research has been focused on developing alternative delivery strategies. Emerging non-invasive routes, including oral, transdermal, and pulmonary delivery, are being investigated, driven by strong patient preference for non-invasive administration, which offers greater convenience, facilitates self-administration, and can significantly enhance adherence and quality of life by reducing the discomfort and burden associated with repeated injections [95–97]. Scientific efforts in drug development, particularly for biopharmaceuticals, are frequently challenged by physiological barriers that significantly impede therapeutic efficacy. The hepatic first-pass effect is a well-known challenge for orally administered drugs, wherein large, protein-based therapeutics are susceptible to extensive proteolytic degradation by gastrointestinal enzymes and subsequent hepatic uptake and lysosomal metabolism before reaching systemic circulation. This pre-systemic elimination drastically reduces bioavailability and requires precise analytical strategies to quantify intact biologics within a complex array of degradation products, often employing LC–MS/MS or immunoassays to discern active drug from inactive fragments across several biological matrices including intestinal contents, portal blood, and liver tissue [98–100]. Similarly, treating central nervous system (CNS) diseases with biopharmaceuticals is severely hindered by the highly restrictive blood–brain barrier (BBB). This specialized neurovascular unit, characterized by tight junctions between endothelial cells and the presence of efflux transporters, effectively limits the passive diffusion of most large, hydrophilic biologics into the brain parenchyma, thereby preventing target engagement at therapeutically relevant concentrations [101–104]. Overcoming these barriers requires innovative formulation and delivery strategies, each demanding rigorous analytical validation to determine drug distribution and concentration at the site of action. Approaches to mitigate the first-pass effect include parenteral administration or advanced oral delivery systems such as enteric-coated formulations or permeation enhancers. Analytical methods employed in these cases include dissolution testing (e.g., using USP apparatus with varying pH media) to assess controlled drug release from enteric-coated systems, and stability-indicating assays like SEC and LC–MS/MS to evaluate biologic integrity and degradation in simulated or ex vivo gastrointestinal environments [105, 106]. For improved systemic exposure after oral delivery, bioanalytical methodologies, including immunoassays (e.g., ELISA) are routinely employed to quantify intact biopharmaceuticals in plasma samples, often utilizing advanced sample preparation techniques to handle the complex matrix [107]. For BBB penetration, strategies range from receptor-mediated transcytosis utilizing specific targeting ligands to transient BBB disruption techniques or direct CNS administration [108]. Each demands sensitive analytical detection of often picomolar concentrations of biopharmaceuticals in complex brain tissue or cerebrospinal fluid samples. Quantitative LC–MS/MS is frequently used for the measurement of biologics in homogenized brain tissue or cerebrospinal fluid samples, often requiring extensive sample cleanup and enrichment [104]. Furthermore, imaging techniques like autoradiography or mass spectrometry imaging provide insights into the spatial distribution of biologics within the brain [109–111]. For in vitro BBB models, transwell systems coupled with fluorescence detection are used to measure permeability coefficients and transcytosis efficiency [108, 112]. Therefore, a comprehensive understanding of these physiological barriers, coupled with sophisticated analytical methodologies, is important for the successful development and quantitative assessment of biopharmaceuticals in challenging biological systems.

### Therapeutic drug monitoring

Therapeutic drug monitoring (TDM) is crucial in optimizing biopharmaceutical therapies by ensuring therapeutic efficacy while minimizing adverse effects. In conditions like inflammatory bowel disease (IBD), TDM facilitates personalized treatment by measuring drug levels of anti-TNFα inhibitors (e.g., adalimumab and infliximab) and detecting anti-drug antibodies (ADAs). Regular monitoring in IBD has been associated with improved clinical response rates and reduced hospitalizations. Up to 73% of patients on infliximab and 35% on adalimumab may develop persistent ADAs, leading to loss of treatment response. Early detection through TDM enables timely dose adjustments or therapy modifications, thereby maintaining disease control [113]. TDM of biopharmaceuticals presents unique analytical and bioanalytical challenges, including assay standardization, point-of-care testing, sensitivity requirements, and sample preparation. Variability among different assays used to measure drug concentrations and ADAs can lead to inconsistent results, thereby complicating clinical decision-making [114–116]. For example, comparative studies of point-of-care and conventional laboratory assays for infliximab and adalimumab quantification have revealed that, despite statistical correlations, these methods exhibit significant non-interchangeability. Specifically, systematic biases at high infliximab concentrations and notable discrepancies within the established therapeutic windows indicate that the choice of assay could directly influence subsequent therapeutic decisions [113]. TDM of biopharmaceuticals employs various analytical techniques, each characterized by distinct sensitivity and specificity levels. ELISA, widely used due to its specificity and sensitivity, enables the detection of low biologic concentrations but is subject to limitations such as cross-reactivity, matrix effects, and reduced ability to detect drug-antibody complexes [115, 117]. In contrast, electrochemiluminescence (ECL) biosensors provide high sensitivity and a broad dynamic range, making them suitable for detecting biologics in complex matrices. Liquid chromatography coupled to tandem mass spectrometry (LC–MS/MS) has demonstrated superior analytical performance. A study comparing ELISA and LC–MS/MS for infliximab detection in serum reported a limit of detection (LOD) of approximately 0.3–0.4 µg mL^−1^ and a lower limit of quantification (LLOQ) of 0.7–1 µg mL^−1^ for LC–MS/MS. These findings highlight the high sensitivity and precision of LC–MS/MS in TDM of biopharmaceuticals [118, 119].

The selection of an appropriate analytical method for TDM of biopharmaceuticals should consider factors such as sensitivity requirements, specificity, throughput, and resource availability. The chosen method must achieve LOD and LLOQ values that align with the therapeutic range of the specific biopharmaceutical and effectively distinguish the target drug from other substances, particularly other endogenous complex biological molecules, in the sample matrix. MS, particularly LC–MS/MS, offers enhanced sensitivity and specificity, facilitating the detection of low-abundance biologics while addressing certain limitations of immunoassays, such as cross-reactivity and the inability to concurrently detect multiple analytes. However, challenges remain regarding automation, suitability for point-of-care testing, complex sample preparation, and the requirement for sophisticated instrumentation [119].

Rapid point-of-care assays, including those based on electrochemical biosensors, offer the advantage of enabling immediate clinical decision-making. However, inconsistencies between these methods and conventional laboratory techniques underscore the requirement for rigorous validation prior to widespread clinical implementation. Furthermore, the absence of standardized point-of-care assays and well-defined regulatory guidelines presents significant barriers to the effective integration of TDM into routine clinical practice [54, 58, 113, 116–118, 120, 121].

Sample preparation is also a crucial factor influencing TDM accuracy. Variables such as sample type (e.g., fresh blood vs. dried blood spots), analyte stability, and pre-analytical factors (e.g., hematocrit levels) can impact assay accuracy. Moreover, the structural complexity and close similarity of biologics to endogenous proteins (e.g., antibodies) present significant challenges in their extraction and purification from biological matrices. Due to their large molecular size, direct measurement of these macromolecules is difficult and often necessitates enzymatic digestion prior to analysis [114, 115, 117, 122].

## SWOT analysis and perspectives for the near future in biopharmaceutical analysis

**Fig. 4:**
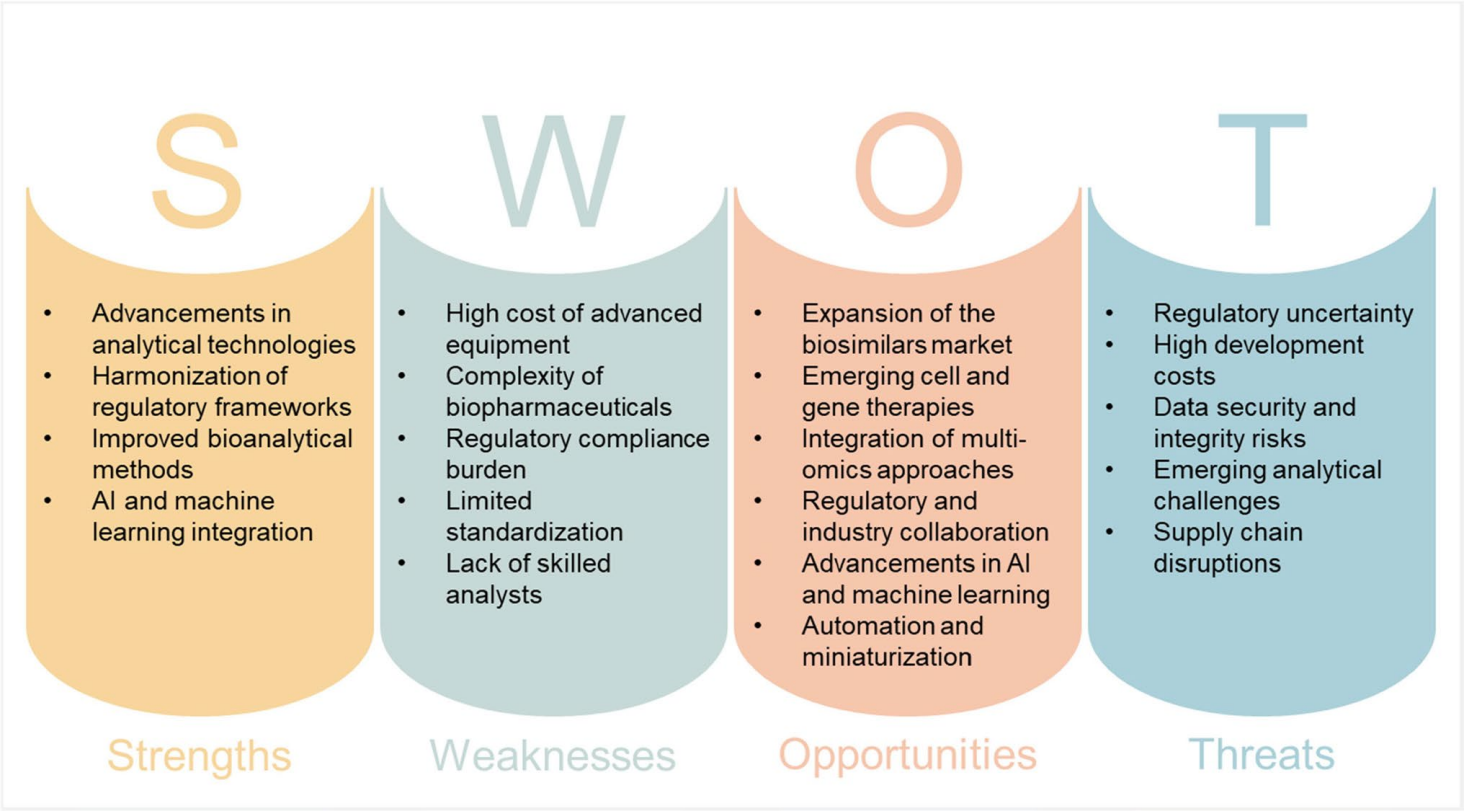
SWOT analysis of future perspectives in biopharmaceutical analysis

Biopharmaceutical analysis is undergoing significant advancements, driven by technological innovations, regulatory requirements, and the need for more precise and efficient analytical and bioanalytical methods. Some of the limitations of the current analytical platforms include the lack of specificity of spectroscopic techniques, the need for validated spectroscopic methods, and the issue of sensors failing when faced with harsh conditions. Additionally, the complexity and cost of automated sampling, chromatographic and MS-based technologies, and the need for validation of multi-attribute monitoring (MAM) methods [40, 123, 124]. The following SWOT analysis (Fig. 4) evaluates the near-future landscape of biopharmaceutical analysis from both analytical and bioanalytical perspectives. One of the major **strengths** in current biopharmaceutical analysis is the continuous advancement of analytical technologies. The integration of NGS, MS-based methods, spectroscopy-based methods, and MAM has significantly improved the accuracy, precision, and specificity of biopharmaceutical characterization [124]. The development of advanced data analytics and machine learning further enhances analytical precision by automating data processing and providing predictive insights [72, 123, 124]. The biopharmaceutical industry is also expanding, with an increasing demand for mAbs, gene therapies, and biosimilars [22, 123, 124]. Biotherapeutics offer improved efficacy and better side-effect profiles compared to traditional small drug molecules, making them an essential focus of pharmaceutical research and development [124]. Furthermore, automation, miniaturization, and artificial intelligence (AI) integration in the existing bioanalytical workflows have streamlined processes, reducing human error and improving reproducibility in analytical testing [52, 123].

Despite these advancements, biopharmaceutical analysis faces several **weaknesses**. The high cost of sophisticated analytical equipment, such as MS, two-dimensional liquid chromatography, and automated sampling systems, presents financial constraints, particularly for smaller industries [124]. Additionally, the complexity of biopharmaceutical products, including their heterogeneity and PTMs, requires extensive analytical methods development, making standardization and reproducibility a challenge [39]. Regulatory compliance also remains a burden, as validation of novel analytical methods (e.g., MAM, NGS, and real-time monitoring tools) is required before implementation [124]. The lack of skilled professionals proficient in advanced analytical techniques, such as chemometrics, MS, and bioinformatics, further complicates the adoption of innovative methodologies [39]. Several **opportunities** exist for the advancement of biopharmaceutical analysis. The rising demand for biosimilars requires improved analytical approaches to demonstrate comparability with originator biopharmaceuticals, thus driving innovation in analytical methodologies [22]. Additionally, emerging cell and gene therapies present new opportunities for bioanalytical development, requiring specialized techniques for potency testing, safety assessments, and product characterization [39, 123]. The integration of multi-omics approaches, combining proteomics, metabolomics, and genomics, enhances analytical insights and supports a deeper understanding of biotherapeutic mechanisms [22]. Collaborative efforts between regulatory agencies and industry stakeholders can also foster the standardization of new analytical platforms and methodologies, expediting their acceptance and implementation [124]. Moreover, advancements in AI and machine learning have the potential to revolutionize data analysis, predictive modeling, and decision-making in biopharmaceutical research [123]. The expected transition to Industry 4.0/5.0 paradigms can also drive the adoption of automation, real-time monitoring, and advanced data analytics. Spectroscopic techniques, for example, offer rapid and in-process analysis supporting real-time monitoring, and the ability to provide molecular fingerprints of samples. Moreover, MAM can substantially decrease the number of samples, the sampling volume, and the required assays [124]. Additionally, more efficient immunocapture systems can offer a cost-effective and more efficient alternative to protein A purification systems [125]. Despite these opportunities, biopharmaceutical analysis faces several **threats**. The long and costly approval processes of biologics, involving extensive preclinical and clinical evaluations, may delay the adoption of novel analytical approaches and impact biopharmaceutical market entry timelines [22]. High development costs associated with new analytical methods can also create financial barriers, limiting access to state-of-the-art methodologies for smaller pharmaceutical and biotechnology companies [124]. Another critical threat is data security and integrity risks, as the increasing reliance on digital tools and automated systems introduces vulnerabilities to cyber threats and data breaches [123]. The emergence of novel biopharmaceuticals presents new analytical challenges that require entirely new characterization strategies, potentially outpacing current analytical capabilities [22]. Furthermore, supply chain disruptions, particularly the lack of critical reagents, columns, and instrumentation, can impact bioanalytical testing and slow down biopharmaceutical production, research, and development [22, 124]. The future of biopharmaceutical analysis presents a dynamic landscape with significant strengths and opportunities, driven by technological advancements, regulatory developments, the growing biopharmaceutical market, and the constant need for personalized treatments. However, challenges such as technical complexities, regulatory obligations, and economic constraints must be addressed to ensure sustainable progress. By leveraging strengths and opportunities while mitigating potential threats, the biopharmaceutical sector can achieve enhanced analytical precision, efficiency, and innovation in biopharmaceutical development and quality control.

## Conclusion

Biopharmaceutical analysis remains a cornerstone of biopharmaceutical drug development and quality assurance, continuously evolving to meet the growing demand for biologics and biosimilars. While advanced analytical methodologies have significantly improved precision and efficiency, challenges persist, including high costs, regulatory constraints, and the inherent complexity of biopharmaceutical products. The heterogeneity of biologics, arising from differences in production processes, host cell systems, and purification strategies, constitutes a significant analytical challenge. Establishing stringent quality attributes and regulatory standards is further complicated by the need to assess protein aggregation, degradation products, and process-related impurities, all of which impact product stability, efficacy, and safety. To address these challenges, cutting-edge bioanalytical technologies, including real-time biosensors, MAM, and process analytical technologies, are increasingly being integrated into biopharmaceutical workflows. These tools enhance in-process monitoring and characterization; however, they require continuous optimization, standardization, and rigorous regulatory validation to ensure compliance. The convergence of AI, machine learning, and high-throughput screening platforms holds promise for transforming biopharmaceutical analysis by streamlining data interpretation, improving predictive modeling, and accelerating quality control processes. Additionally, the adoption of real-time release testing and automation-driven manufacturing strategies is expected to reduce approval timelines and enhance production efficiency, thereby facilitating the rapid development of novel biopharmaceuticals and biosimilars. As the field advances, strategic investments in next-generation analytical platforms, digitalization, and workforce training are essential to overcoming existing barriers. Collaboration between regulatory agencies, industry stakeholders, and academia is crucial for the standardization of analytical approaches, ensuring data integrity, and fostering innovation. Ultimately, the continued progress of biopharmaceutical analysis will drive improvements in therapeutic precision, manufacturing efficiency, and global healthcare accessibility, opening the way for the next era of biopharmaceuticals and precision medicine.

## Data Availability

Data will be made available on request.
